# Sex Differences in Temporal Trends in Hospitalizations and In-Hospital Mortality in Patients with Sarcoidosis in Spain from 2001 to 2020

**DOI:** 10.3390/jcm11185367

**Published:** 2022-09-13

**Authors:** Belén López-Muñiz Ballesteros, Concepción Noriega, Ana Lopez-de-Andres, Rodrigo Jimenez-Garcia, Jose J. Zamorano-Leon, David Carabantes-Alarcon, Javier de Miguel-Díez

**Affiliations:** 1 Respiratory Department, Hospital Universitario Infanta Leonor, 28031 Madrid, Spain; 2 Department of Nursery and Physiotherapy, Faculty of Medicine and Health Sciences, University of Alcalá, Alcalá de Henares, 28801 Madrid, Spain; 3 Department of Public Health & Maternal and Child Health, Faculty of Medicine, Universidad Complutense de Madrid, 28040 Madrid, Spain; 4 Respiratory Department, Hospital General Universitario Gregorio Marañón, Instituto de Investigación Sanitaria Gregorio Marañón (IiSGM), Faculty of Medicine, Universidad Complutense de Madrid, 28007 Madrid, Spain

**Keywords:** sarcoidosis, sex, hospitalizations, in-hospital mortality, Spain

## Abstract

(1) Background: We aimed to analyze temporal trends in hospitalization and in-hospital mortality (IHM) in patients with sarcoidosis in Spain from 2001–2020. (2) Methods: Using the Spanish National Hospital Discharge Database, we included patients (aged ≥ 20 years) hospitalized with a sarcoidosis code in any diagnostic field. (3) Results: We included 44,195 hospitalizations with sarcoidosis (56.34% women). The proportion of women decreased over time, from 58.76% in 2001 and 2002 to 52.85% in 2019 and 2020 (*p* < 0.001). The crude rates per 100,000 inhabitants increased by 4.02% per year among women and 5.88% among men. These increments were confirmed using Poisson regression analysis, which yielded an IRR of 1.03; 95% CI 1.01–1.04 for women and 1.04; 95% CI 1.02–1.06 for men. During the study period, no significant sex differences in IHM were recorded. Older age, COVID-19, respiratory failure, and the need for mechanical ventilation were independent predictors of IHM in men and women hospitalized with sarcoidosis, with IHM remaining stable over time. (4) Conclusions: The number of hospital admissions among patients with sarcoidosis in Spain increased threefold from 2001 to 2020. Although the incidence rates were higher in women, the trend followed that the incidence rates between sexes became closer. IHM was similar among men and women, with no significant change over time in either sex after multivariable analysis.

## 1. Introduction

Sarcoidosis is a rare, multisystem disease of unknown cause. It can affect any organ, although the lung is the most frequent site. Sarcoidosis is characterized by the formation of non-necrotizing granulomas in the affected organ [1].

The prevalence of this disease is higher in the third and fourth decades of life in men and in the fifth and sixth decades in women, with considerable variations from one country to another. For example, it is estimated that the prevalence of sarcoidosis is 11.5 per 100,000 inhabitants in Sweden compared to 8.1 per 100,000 inhabitants in the United States. The disease is much less frequent in Asia; for example, in South Korea the prevalence is estimated to be 0.5–1.3 per 100,000 inhabitants [2,3,4,5].

Studies report an increase in the number of hospital admissions for any reason in patients with a previous diagnosis of sarcoidosis. A study analyzing trends in hospitalizations for this disease between 1979 and 2000 revealed an increase over time, from 108 per 100,000 hospitalizations in 1979 to 146 per 100,000 hospitalizations in 2000 [6]. This upward trend has been maintained in subsequent years. Another, more recent study also found an increase in the number of admissions, from 138 per 100,000 hospitalizations in 2005 to 175 per 100,000 hospitalizations in 2014 [7]. This trend arose owing to better knowledge of the disease over time, the progressive increase in the frequency of comorbidities, and the more widespread use of immunosuppressive therapies, as well as the complications resulting from their administration. The main risk factors for the increase over time in both admissions and readmissions are advanced age, respiratory comorbidity, and disease severity [8].

Mortality in patients with sarcoidosis has also been increasing over time, ranging from 1% to 8% depending on disease severity, systemic manifestations, age, sex, race, and geographic distribution [8]. Nevertheless, reported data are contradictory. In the United States, Swigris et al. [9] reported a mortality rate of 4.32 per 1,000,000 inhabitants between 1988 and 2007. The cause of death was the disease itself in 58.8% of cases. Most of those who died were women, patients older than 55 years, and African American individuals. In the series reported by Duncan et al. [10], the mortality rate for patients with sarcoidosis during the period from 1979–1983 was 3.3/1,000,000 inhabitants compared to 4.1/1,000,000 inhabitants in the period from 2001–2008. However, in the study by Patel et al. [7], in-hospital mortality (IHM) decreased from 6.5 to 4.9 per 100 patients admitted for sarcoidosis between 2005 and 2014.

Our main objective was to describe and analyze temporal trends in hospitalization and IHM in patients with sarcoidosis in Spain from 2001 to 2020. We also aimed to assess sex differences in the rates of hospitalizations, clinical characteristics, and hospital outcomes in patients with sarcoidosis who were hospitalized. Finally, we identified which comorbidities and procedures were independent predictors of IHM in women and men with sarcoidosis that were admitted to hospitals.

## 2. Materials and Methods

We analyzed data for the period from 2001 to 2020 from the Spanish National Hospital Discharge Database (SNHDD), which belongs to and is managed by the Spanish Ministry of Health (SMH). Since all private and public hospitals provide their data to the SNHDD, it is estimated that over 98% of hospitalizations are recorded, that is, more than 4 million annually [11].

Between 1992 and December 2015, diseases in the SNHDD were coded according to the International Classification of Diseases, Ninth Revision, Clinical Modification (ICD-9-CM); since then, coding has been based on the International Classification of Diseases, Tenth Revision, Clinical Modification (ICD-10-CM) [11,12].

### 2.1. Study Population

We included all hospitalized patients aged 20 years or over who had a sarcoidosis code in any diagnostic position (ICD-9-CM code 135 and ICD-10-CM code D86XX) in Spain from 2001–2020. The SNHDD covered up to 14 diagnoses with the ICD-9-CM and up to 20 with the ICD-10-CM [12]. We excluded patients with missing data for age (*n* = 14), sex (*n* = 23), dates of admission (*n* = 3), discharge (*n* = 3), and discharge destination (*n* = 37) (this variable included four options: home, another health or social institution, voluntary discharge, and death in the hospital). Based on these criteria, the study population finally comprised 44,195 patients, 251 of whom (0.56%) were excluded with no imputation of missing data.

### 2.2. Study Variables

Our main outcome variables were the incidence of hospitalizations with sarcoidosis per 100,000 inhabitants, the length of hospital stay (LOHS), and IHM. All analyses were conducted separately for women and men, and the results were compared to assess possible sex differences.

We specifically identified patients with pulmonary sarcoidosis using the methods suggested by Alqalyoobi et al. [13]. Study covariates included age and the Charlson Comorbidity Index (CCI), which was calculated as a continuous variable for each patient following the recommendations of Quan et al. [14]. The specific respiratory conditions analyzed were pneumonia, COVID-19 (only the year 2020), respiratory failure, chronic obstructive pulmonary disease (COPD), asthma, acute pulmonary embolism (PE), pulmonary hypertension, obstructive sleep apnea (OSA), and the use of oxygen prior to hospital admission (this code refers to chronic oxygen supplementation). Non-respiratory diagnoses included gastroesophageal reflux disease, scleroderma, rheumatoid arthritis, dermatomyositis and polymyositis, obesity, and smoking. We also extracted data on in-hospital procedures, such as invasive mechanical ventilation (IMV), non-invasive mechanical ventilation (NIMV), and lung transplant. The ICD-9-CM and ICD-10-CM codes used to extract these diagnoses and procedures are shown in Table S1.

### 2.3. Statistical Analysis

The incidence of hospitalizations was calculated using population data obtained from the Spanish National Statistics Institute [15]. Joinpoint log linear regression was applied to analyze temporal trends in crude incidence and to estimate the annual percentage change (APC) for women and men; this method was also used to estimate IHM.

Poisson regression models were constructed to assess age-adjusted temporal trends and to compare incidence rates between women and men. The incidence rate ratio (IRR) was obtained with its 95% confidence interval (95% CI).

Continuous variables were reported as a mean with standard deviation (SD) or median with interquartile range (IQR). Categorical variables were reported as counts and percentages.

The trend was analyzed using a linear regression t-test for means, Jonckheere–Terpstra test for medians, and Cochran–Mantel–Haenszel statistic or Cochran–Armitage test for categorical variables.

Continuous variables were compared using the t-test or Wilcoxon rank sum test for mean and median, respectively. Categorical variables were compared using the chi-square test.

We constructed three multivariable logistic regression models to identify which study variables were independently associated with IHM after hospitalization with sarcoidosis among women, men, and both sexes. The variables included in these models were age, sex, year of hospital admission, and all those clinical variables that were significantly associated (*p* < 0.10) with IHM in the bivariate analysis.

The analysis was performed using Joinpoint Regression Program, version 4.0.4 (Statistical Research and Applications Branch, National Cancer Institute, Bethesda, MD, USA) and Stata version 14 (Stata, College Station, TX, USA).

### 2.4. Ethics

When the SNHDD database was provided to us by the Spanish Ministry of Health, all patient data had been de-identified. Therefore, according to Spanish law, institutional review board approval for this study was not required. The application form for the SNHDD can be downloaded online [16].

## 3. Results

### 3.1. Baseline Characteristics and Hospitalization Trends

The distribution according to sex and age of the 44,195 hospitalizations with sarcoidosis in Spain between 2001 and 2020 can be seen in Table 1. The mean age was 59.99 years (SD 16.30), and 24,901 were women (56.34%). Women were older than men (62.23 vs. 57.09 years; *p* < 0.001) and the mean age increased significantly over time for both sexes. The proportion of women decreased from 58.76% in 2001–2002 to 52.85% in 2019–2020 (*p* < 0.001).

The total number of cases increased constantly over time for both sexes, from 1472 women and 1053 men in 2001–2002 to 3469 and 3095 in 2019–2020, respectively.

**Figure 1:**
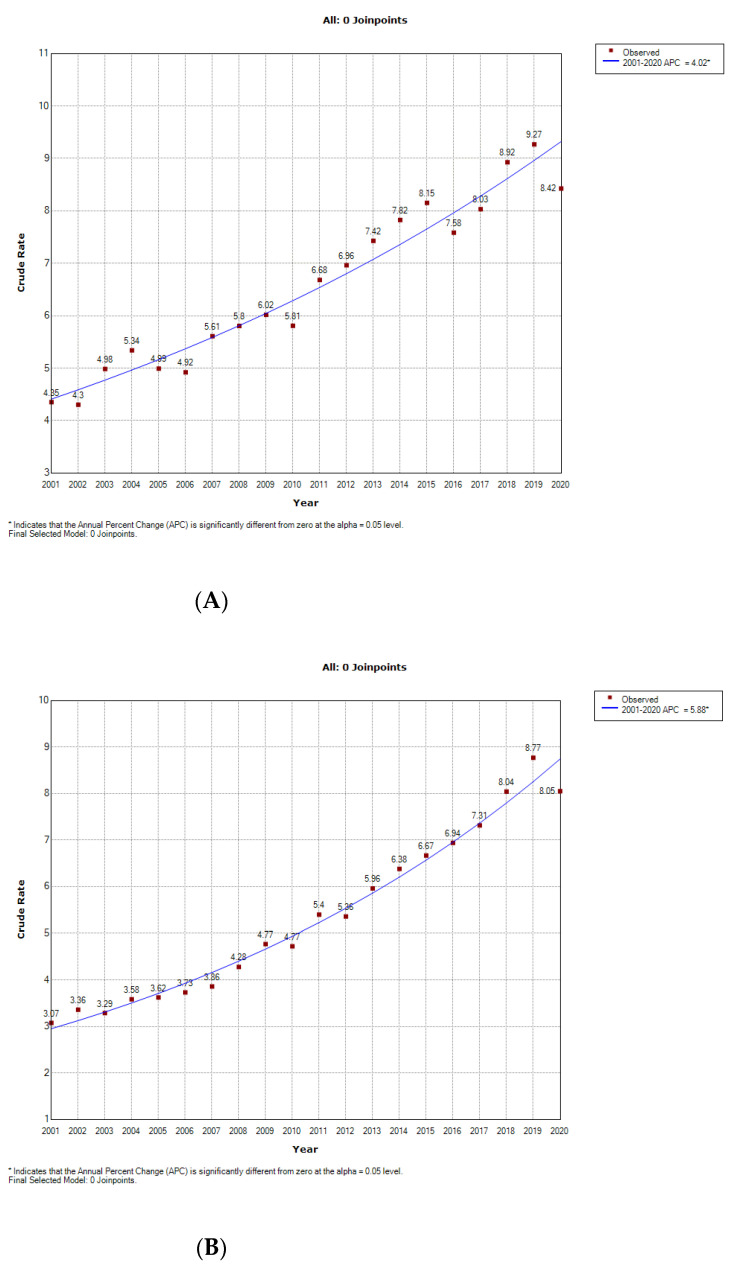
Joinpoint regression to assess time trend in the incidence per 100,000 inhabitants of hospitalizations with sarcoidosis in Spain from 2001 to 2020, according to sex. (**A**) Women. (**B**) Men.

Analysis of temporal trends from 2001 to 2020, according to the crude rates per 100,000 inhabitants and based on Joinpoint regression for women (Figure 1A) and men (Figure 1B), revealed APC values of 4.02% and 5.88%, respectively. These increments were confirmed by age-adjusted Poisson regression analysis, which yielded an IRR of 1.03 (95% CI 1.01–1.04) for women and 1.04 (95% CI 1.02–1.06) for men. For the entire period, and after adjusting for age, the incidence rate of hospitalizations among women was slightly but significantly higher than the incidence for men (IRR 1.02; 95% CI 1.00–1.03).

### 3.2. Trends in Comorbidities, Procedures, and In-Hospital Outcomes

Women hospitalized with sarcoidosis had over twice as many comorbid conditions, according to the CCI, during the last time-period described (1.1 in 2017–2020) than during the first one (0.53 in 2001–2004) (Table 2).

The proportion of women with pulmonary sarcoidosis rose from 36.23% in 2001–2004 to 50.05% in 2017–2020 (*p* < 0.001). Furthermore, all the respiratory conditions analyzed became significantly more frequent over time. Analysis of discharge codes for the period from 2017–2020 revealed that 9.64% of women had respiratory failure, 8.53% asthma, 7.09% pulmonary hypertension, 6.17% pneumonia, and 5.19% OSA.

The prevalence of obesity, smoking, use of oxygen prior to hospital admission, and the need for NIMV during hospitalization also increased over time. The number of patients with a code for lung transplant among adults hospitalized with sarcoidosis was very low during the study period, ranging from 3 to 11 for the two periods analyzed. No significant change overtime was detected. (Figure S1).

The median LOHS decreased from 7 days in 2001–2004 to 6 days in 2017–2020 (*p* < 0.001).

A statistically significant linear trend was observed in the crude IHM for women with sarcoidosis who were hospitalized between 2001 and 2020 (*p* = 0.005), with later years being associated with higher mortality (Table 2). In 2020, COVID-19 was recorded in 155 women (2.3%).

The temporal trend for comorbidities in inpatient men with sarcoidosis is very similar to that of women (Table 3). The CCI value doubled, from 0.65 in 2001–2004 to 1.25 in 2017–2020. The frequency of respiratory conditions and the need for oxygen prior to hospital admission increased over time, except in the case of asthma, which remained stable. The frequency of obesity (4.07% vs. 7.75%; *p* < 0.001), smoking (22.86% vs. 24.75%; *p* < 0.001), and NIMV (0.68% vs. 1.85%) also rose from the first to the last time period.

Regarding hospital outcomes, the median LOHS decreased from 8 to 6 days (*p* < 0.001), and the crude IHM increased from 2.61% in 2001–2004 to 4.98 in 2017–2020 (*p* < 0.001). COVID-19 was detected in 2.18% of men (128).

### 3.3. Sex Differences in Clinical Conditions and Hospital Outcomes

After analyzing the distribution according to the study, we observed that women hospitalized with sarcoidosis had a higher prevalence of respiratory failure, asthma, pulmonary hypertension, obesity, and the need for oxygen prior to hospital admission. In men, on the other hand, higher values were observed for pulmonary sarcoidosis (44.47% vs. 39.69%; *p* < 0.001), pneumonia, COPD, OSA, smoking, and IMV (Table 4).

No significant differences were recorded for LOHS or IHM during the study period (4.13% for men vs. 4.06% for women: *p* = 0.694).

The IHM according to study variables is shown in Table 4. For both sexes, the highest IHM was found among patients requiring IMV or NIMV. All the respiratory conditions described were associated with frequent IHM, except for asthma, which was associated with low IHM in women (2.93%) and men (1.95%). The joinpoint log linear regression for IHM year by year is shown in Supplementary Figure S2. The IHM among women with sarcoidosis varied between 2.96% in 2003 and 6.02% in 2020, with a significant APC of 1.82%. For men, the lowest and highest percentages were 2.19% and 5.91% for the same years, with a lower APC of 0.13% (*p* < 0.05).

### 3.4. Predictors of In-Hospital Mortality

After adjustment for all the covariates using multivariable logistic regression, we found that, for both sexes, older age, COVID-19, respiratory failure, and the need for IMV or NIMV were independent predictors of IHM in patients with sarcoidosis who were hospitalized (Table 5). However, asthma and obesity were associated with reduced mortality in women and men. Pneumonia and acute PE were also independent predictors of IHM, although only among women.

Analysis of the entire database revealed that, in addition to all the predictors already mentioned, the need for oxygen prior to hospital admission and male sex (OR 1.24; 95% CI 1.11–1.38) increased the risk of dying among inpatients with sarcoidosis.

Finally, when the temporal trend in IHM was analyzed after adjusting for all covariates, we observed no significant change in the IHM over time for either sex.

## 4. Discussion

We recorded 44,195 hospitalizations for sarcoidosis in Spain between 2001 and 2020. Analysis by period revealed that the total number of admissions had tripled over time in both sexes, rising from 1472 in women and 1053 in men during 2001–2002 to 3469 and 3095, during 2019–2020, respectively. This trend represents increases in hospital admissions of 4.02% in women and 5.88% in men between 2001 and 2020, as reported in other series [17,18]. Alqalyoobi et al. [13] observed that the number of hospitalizations for sarcoidosis increased from 258.5 per 1,000,000 hospitalizations in 2007 to 705.7 per 1,000,000 hospitalizations in 2018, although the authors only evaluated patients with pulmonary sarcoidosis. We also observed an increase in hospitalizations in patients with pulmonary sarcoidosis, both in men and women. In any case, the increase in the number of admissions in patients with a previous diagnosis of sarcoidosis is mainly due to other conditions, rather than to sarcoidosis itself [18]. Possible explanations for this upward trend include the following: patients are admitted more frequently for invasive procedures, sarcoidosis is associated with a high number of comorbidities, knowledge of the disease has improved, and immunosuppressive treatment is more frequent, with the potential risk of associated complications [19,20,21].

The mean age of the patients admitted was 59.9 years, somewhat higher than the age at diagnosis [2,3]. This age has been increasing in both sexes over time. Regarding sex, the number of admissions was higher for women than for men (56.3%), although the difference decreased progressively. Thus, during 2001–2002, 58.76% of all admissions were women, compared to 52.86% in 2019–2020. These results are similar to those reported by Fidler et al. [2], who analyzed trends in hospitalizations of patients with sarcoidosis in Ontario, Canada between 1996 and 2015, recording 18,550 patients admitted with a previous diagnosis of sarcoidosis. In this population, the admission rate adjusted for any cause of hospitalization fell from 206.4 to 152.1 per 1000 cases between 1996 and 2015, with this drop being greater in women than in men (a reduction in hospitalizations of 85% in women compared to 72% in men) and in younger patients (a 91% drop in those aged 26–35 years) [22].

Sarcoidosis is more prevalent in Black people and women [4,23,24,25,26]. In our study, an increase in the number of admissions for any reason was observed in women with a previous diagnosis of sarcoidosis, tending to approach that of men over time. This change in the trend could be justified by the fact that the prevalence of sarcoidosis differs little by sex with age and is almost similar in older men and women, in whom the number of admissions has also increased in recent years [23,25]. In addition, advanced age implies a worse prognosis for sarcoidosis, with not only a higher rate of hospital admissions, but also an increase in mortality. It is noteworthy that, although sarcoidosis is more prevalent among younger individuals, the A Case Control Etiologic Study of Sarcoidosis (ACCESS) registry identified that 30% of patients were over 50 years of age, and different series have suggested that there could be a second peak in incidence between 50 and 65 years [9,26,27]. In any case, the increase in mortality in older patients could be justified by the increase in comorbidities in this group, who also tend to more frequently present dyspnea, anxiety, obstructive respiratory diseases, cognitive impairment, and signs and symptoms that could cause sarcoidosis to be confused with other diseases, thus making it difficult to diagnose and manage the disease in this population [28,29].

We detected an increase in comorbidities associated with sarcoidosis over time, with no differences between the sexes. Thus, the CCI increased from 0.65 in the period from 2001–2004 to 1.1 in the period from 2017–2020. Previous studies have shown that because sarcoidosis is a systemic disease, it is more likely to be associated with comorbidities, thus implying an increase in morbidity and mortality and, therefore, a higher number of hospital admissions [22]. The most frequent comorbidities associated with sarcoidosis include cardiovascular disease, respiratory disease, and cancer [30,31]. In the case of women, the comorbidities most frequently associated with sarcoidosis in our study were asthma, obesity, pulmonary hypertension, and respiratory failure. In men, the most common comorbidities were COPD, OSA, and pneumonia.

The presence of respiratory comorbidities, except for asthma, was associated with higher IHM, which was similar in both sexes and higher in patients with severe respiratory failure that required treatment with NIMV or orotracheal intubation. These two situations, together with advanced age and the presence of pneumonia or PE, were associated with a higher risk of dying. Various studies have shown that the main risk factors for mortality in patients with sarcoidosis are age, extent of fibrosis, pulmonary hypertension, and, mainly, progression of respiratory failure [13,32].

Patients with respiratory diseases secondary to or associated with sarcoidosis have a worse prognosis. In our series, the prevalence of PE among patients with sarcoidosis was 1.05% in men and 1.12% in women, with a mortality rate of 8.91% in men and 13.26% in women. Although the association between PE and sarcoidosis has been described in different studies and the pathogenic mechanism remains unknown, an increase in mortality has been reported in affected patients [21,33]. As in other series, mortality was higher for patients who were admitted with acute respiratory failure and required mechanical ventilation (IMV and NIMV), with no differences between the sexes. In this sense, a previous study showed that patients admitted for acute respiratory failure had a 13-fold higher mortality, reaching 26-fold in those who required mechanical ventilation [13].

Analysis of crude IHM revealed an increase over time, from 2.96% in women and 2.19% in men in 2003 compared to 6.02% and 5.91% in 2020, respectively. The explanation for these results, in addition to the risk factors that worsen the prognosis that we analyzed previously (severe respiratory failure, advanced age, and PE), could be that in the year in 2020, the pandemic caused by the SARS-CoV-2 virus has been shown to be an independent factor for IHM in patients with sarcoidosis. In our study, this infection was detected in 2.18% of men and 2.3% of women, with an overall mortality of 15.48% in both sexes. In published series on COVID-19 and sarcoidosis, it has been shown that the risk of presenting a severe form of the disease is similar to that of other immune-mediated diseases, with the highest-risk patients being those receiving immunosuppressive treatment, those with more associated comorbidities, older patients, and those who are in a situation of previous respiratory insufficiency or have worse lung function. However, these associations do not seem to vary according to the sarcoidosis phenotype or the organ affected [34,35,36,37]. In any case, when the temporal trend in the IHM was analyzed in our study after adjusting for all covariates, we observed no significant change in IHM in men or women.

Our study has several limitations. First, data were obtained from an administrative database in which diseases and procedures are coded using ICD-9-CM or ICD-10-CM, depending on the year of study; therefore, changes in coding practices over time may have occurred. Second, we have not described the site of extrapulmonary sarcoidosis. The reason for this is that the ICD-9, used by the SNHDD from 2001 to 2015, has a single code for sarcoidosis and does not distinguish which organs are affected by this disease. However, Alqalyoobi et al. used an algorism, using other complementary ICD-9 code (Table S1), to identify patients with pulmonary sarcoidosis, that we have used for the period from 2001–2015 [13]. To our knowledge there are no algorisms that can be used to identify patients with sarcoidosis affecting other organs using the ICD-9. Third, given the characteristics of our database, we do not have information on the sarcoidosis stage because the SNHDD only collects diagnosis and procedures but not stages of disease. Furthermore, we do not have the results of the chest radiography of the patients included, so it is also not possible to establish the stage this way. Fourth, since our database does not include the treatments received by patients, immunosuppressive treatment of sarcoidosis can be a cause of morbidity and mortality during hospital admissions [38]. Fifth, the ICD-9 and ICD-10 codes do not allow the identification of exacerbations of sarcoidosis or other interstitial lung diseases. Sixth, the information collected by the SNHDD, does not include the main reason for mortality. Therefore, there is no definite and reliable way to ascertain that the death was related to sarcoidosis. Rather, we used the “overall” IHM as provided by the SNHDD database. Finally, the total number of patients with sarcoidosis that had to be excluded for missing data on relevant variables was only 80. This represents under 0.2% of the study population so, in our opinion, the bias that could result from this missing data, if any, would be of a very small magnitude. Despite these limitations, the SNHDD has the advantage of being part of the Spanish National Health System and covers almost 100% of admissions in Spain [11]. In addition, Spain is a large country with a public health system that provides complete and free medical coverage to the entire population. Therefore, the fact that patients come from a wide variety of socioeconomic categories improves the external validity of our results.

## 5. Conclusions

The number of hospital admissions in patients with a previous diagnosis of sarcoidosis increased threefold from 2001 to 2020 in Spain. Although the incidence was higher in women, the trend followed similar values in both sexes. Furthermore, after adjustment for all covariates, IHM did not significantly change over time for either sex. Factors independently associated with IHM in men and women admitted with sarcoidosis included, in addition to SARS-CoV-2 infection, age and the need for mechanical ventilation.

## Figures and Tables

**Table 1:** Number of hospitalizations of adults with sarcoidosis in Spain from 2001 to 2020, according to sex and age.

Sex		2001/02	2003/04	2005/06	2007/08	2009/10	2011/12	2013/14	2015/16	2017/18	2019/20	*p*
Men	Age, mean (SD)	48.97 (16.06)	52.28 (16.77)	52.84 (16.72)	54.04 (16.44)	54.51 (16.5)	56.88 (16.16)	57.75 (15.65)	58.86 (15.46)	60.6 (15.03)	61.36 (15.17)	<0.001
	20–44 years, *n* (%)	460 (44.53)	437 (37.87)	459 (35.72)	486 (33.15)	534 (30.51)	530 (26.62)	515 (22.75)	514 (20.81)	484 (17.37)	512 (16.54)	<0.001
	45–59 years, *n* (%)	260 (25.17)	287 (24.87)	331 (25.76)	400 (27.29)	505 (28.86)	544 (27.32)	681 (30.08)	732 (29.64)	786 (28.21)	828 (26.75)	
	60–74 years, *n* (%)	261 (25.27)	313 (27.12)	352 (27.39)	387 (26.4)	470 (26.86)	578 (29.03)	701 (30.96)	779 (31.54)	974 (34.96)	1081 (34.93)	
	≥75 years, *n* (%)	52 (5.03)	117 (10.14)	143 (11.13)	193 (13.17)	241 (13.77)	339 (17.03)	367 (16.21)	445 (18.02)	542 (19.45)	674 (21.78)	
	All age groups, *n* (%)	1033 (100)	1154 (100)	1285 (100)	1466 (100)	1750 (100)	1991 (100)	2264 (100)	2470 (100)	2786 (100)	3095 (100)	NA
Women	Age, mean (SD)	56.61 (16.38)	58.1 (16.09)	59.28 (16.21)	60.64 (16.17)	60.19 (16.12)	62.55 (15.99)	62.86 (15.94)	64.14 (15.36)	65.01 (15.36)	65.58 (15.07)	<0.001
	20–44 years, *n* (%)	364 (24.73)	403 (22.09)	383 (21.17)	436 (20.36)	442 (19.43)	425 (16.04)	456 (15.44)	385 (12.7)	411 (12.55)	386 (11.13)	<0.001
	45–59 years, *n* (%)	403 (27.38)	479 (26.26)	443 (24.49)	427 (19.94)	545 (23.96)	622 (23.47)	713 (24.14)	705 (23.26)	674 (20.57)	722 (20.81)	
	60–74 years, *n* (%)	491 (33.36)	664 (36.4)	640 (35.38)	804 (37.55)	780 (34.29)	873 (32.94)	965 (32.67)	1085 (35.8)	1195 (36.48)	1192 (34.36)	
	≥75 years, *n* (%)	214 (14.54)	278 (15.24)	343 (18.96)	474 (22.14)	508 (22.33)	730 (27.55)	820 (27.76)	856 (28.24)	996 (30.4)	1169 (33.7)	
	All age groups *n* (%)	1472 (100)	1824 (100)	1809 (100)	2141 (100)	2275 (100)	2650 (100)	2954 (100)	3031 (100)	3276 (100)	3469 (100)	NA
Both sexes	Age, mean (SD)	53.46 (16.68)	55.8 4(16.6)	56.61 (16.73)	57.96 (16.6)	57.72 (16.53)	60.12 (16.31)	60.64 (16.01)	61.77 (15.62)	62.99 (15.37)	63.59 (15.27)	<0.001
	20–44 years, *n* (%)	824 (32.89)	840 (28.21)	842 (27.21)	922(25.56)	976(24.25)	955 (20.58)	971 (18.61)	899 (16.34)	895 (14.76)	898 (13.68)	<0.001
	45–59 years, *n* (%)	663 (26.47)	766 (25.72)	774 (25.02)	827(22.93)	1050(26.09)	1166 (25.12)	1394 (26.72)	1437 (26.12)	1460 (24.08)	1550 (23.61)	
	60–74 years, *n* (%)	752 (30.02)	977 (32.81)	992 (32.06)	1191(33.02)	1250(31.06)	1451 (31.26)	1666 (31.93)	1864 (33.88)	2169 (35.78)	2273 (34.63)	
	≥75 years, *n* (%)	266 (10.62)	395 (13.26)	486 (15.71)	667(18.49)	749(18.61)	1069 (23.03)	1187 (22.75)	1301 (23.65)	1538 (25.37)	1843 (28.08)	
	All age groups *n* (%)	2505 (100)	2978 (100)	3094 (100)	3607(100)	4025(100)	4641 (100)	5218 (100)	5501 (100)	6062 (100)	6564 (100)	NA

*p* value for time trend (linear regression t-test for age and Cochran–Mantel–Haenszel statistic for age groups). NA: not applicable.

**Table 2:** Trends in the comorbidities, procedures, and in-hospital outcomes among women hospitalized with sarcoidosis in Spain from 2001 to 2020.

Variable	2001/04	2005/08	2009/12	201/-16	2017/20	*p*
Charlson Comorbidity Index, mean (SD)	0.65 (0.53)	0.77 (0.61)	0.92 (0.88)	1.03 (0.94)	1.1 (1.01)	<0.001
Pulmonary sarcoidosis, *n* (%)	1194 (36.23)	1369 (34.66)	1811 (36.77)	2134 (35.66)	3376 (50.05)	<0.001
Pneumonia, *n* (%)	121 (3.67)	178 (4.51)	240 (4.87)	314 (5.25)	416 (6.17)	<0.001
COVID 19, *n* (%)	NA	NA	NA	NA	155 (2.3)	<0.001
Respiratory failure, *n* (%)	401 (12.17)	658 (16.66)	821 (16.67)	1007 (16.83)	1325 (19.64)	<0.001
COPD, *n* (%)	65 (1.97)	91 (2.3)	145 (2.94)	181 (3.02)	235 (3.48)	<0.001
Asthma, *n* (%)	228 (6.92)	293 (7.42)	412 (8.37)	502 (8.39)	576 (8.54)	0.021
Acute pulmonary embolism, *n* (%)	21 (0.64)	35 (0.89)	64 (1.3)	59 (0.99)	100 (1.48)	0.001
Pulmonary hypertension *n* (%)	105 (3.19)	244 (6.18)	350 (7.11)	461 (7.7)	478 (7.09)	<0.001
Obstructive sleeping apnea *n* (%)	NA	7 (0.18)	67 (1.36)	167 (2.79)	350 (5.19)	<0.001
Gastroesophageal reflux disease *n* (%)	22 (0.67)	35 (0.89)	47 (0.95)	84 (1.4)	162 (2.4)	<0.001
Scleroderma, *n* (%)	5 (0.15)	8 (0.2)	11 (0.22)	14 (0.23)	34 (0.5)	0.004
Rheumatoid Arthritis *n* (%)	20 (0.61)	54 (1.37)	61 (1.24)	89 (1.49)	139 (2.06)	<0.001
Dermatomyositis and Polymyositis, *n* (%)	0 (0)	3 (0.08)	3 (0.06)	3 (0.05)	5 (0.07)	0.632
Obesity, *n* (%)	227 (6.89)	317 (8.03)	495 (10.05)	656 (10.96)	695 (10.3)	<0.001
Tobacco use, *n* (%)	164 (4.98)	240 (6.08)	377 (7.65)	531 (8.87)	494(7.32)	<0.001
Oxygen prior to hospital admission, *n* (%)	36 (1.09)	137 (3.47)	277 (5.62)	310 (5.18)	488(7.23)	<0.001
IMV, *n* (%)	30 (0.91)	50 (1.27)	45 (0.91)	64 (1.07)	80(1.19)	0.385
NIMV, *n* (%)	18 (0.55)	43 (1.09)	57 (1.16)	130 (2.17)	105(1.56)	<0.001
LOHS, median (IQR)	7 (9)	7 (9)	6 (8)	6 (7)	6(7)	<0.001
IHM, *n* (%)	117 (3.55)	159 (4.03)	179 (3.63)	231 (3.86)	324(4.8)	0.005

COPD: chronic obstructive pulmonary disease. NA: not available. BMI: body mass index. IMV: invasive mechanical ventilation. NIMV: non-invasive mechanical ventilation. LOHS: length of hospital stay. IHM: in-hospital mortality. *p* value for time trend (Cochran–Armitage tests for binary variables and Jonckheere–Terpstra test for the LOHS).

**Table 3:** Trends in the comorbidities, procedures, and in-hospital outcomes among men hospitalized with sarcoidosis in Spain from 2001 to 2020.

Variable	2001/04	2005/08	2009/12	201/-16	2017/20	*p*
Charlson Comorbidity Index, mean (SD)	0.65 (0.55)	0.87 (0.72)	0.96 (0.84)	1.1 (0.98)	1.25 (1.11)	<0.001
Pulmonary sarcoidosis, *n* (%)	891 (40.74)	1090 (39.62)	1520 (40.63)	1925 (40.66)	3155 (53.65)	<0.001
Pneumonia, *n* (%)	91 (4.16)	131 (4.76)	200 (5.35)	274 (5.79)	416 (7.07)	<0.001
COVID 19, *n* (%)	NA	NA	NA	NA	128 (2.18)	<0.001
Respiratory failure, *n* (%)	262 (11.98)	347 (12.61)	534 (14.27)	693 (14.64)	1103 (18.76)	<0.001
COPD, *n* (%)	115 (5.26)	188 (6.83)	296 (7.91)	410 (8.66)	675 (11.48)	<0.001
Asthma, *n* (%)	53 (2.42)	90 (3.27)	117 (3.13)	153 (3.23)	201 (3.42)	0.259
Acute pulmonary embolism, *n* (%)	7 (0.32)	13 (0.47)	39 (1.04)	53 (1.12)	90 (1.53)	<0.001
Pulmonary hypertension *n* (%)	75 (3.43)	125 (4.54)	168 (4.49)	222 (4.69)	293 (4.98)	0.061
Obstructive sleeping apnea *n* (%)	0 (0)	7 (0.25)	116 (3.1)	266 (5.62)	521 (8.86)	<0.001
Gastroesophageal reflux disease *n* (%)	10 (0.46)	16 (0.58)	46 (1.23)	48 (1.01)	110 (1.87)	<0.001
Scleroderma, *n* (%)	4 (0.18)	2 (0.07)	5 (0.13)	3 (0.06)	18 (0.31)	0.020
Rheumatoid Arthritis *n* (%)	18 (0.82)	13 (0.47)	21 (0.56)	36 (0.76)	48 (0.82)	0.297
Dermatomyositis and Polymyositis, *n* (%)	7 (0.32)	2 (0.07)	3 (0.08)	8 (0.17)	11 (0.19)	0.160
Obesity, *n* (%)	89 (4.07)	131 (4.76)	209 (5.59)	315 (6.65)	456 (7.75)	<0.001
Tobacco use, *n* (%)	500 (22.86)	684 (24.86)	1177 (31.46)	1332 (28.14)	1456 (24.76)	<0.001
Oxygen prior to hospital admission, *n* (%)	3 (0.14)	73 (2.65)	134 (3.58)	221 (4.67)	400 (6.8)	<0.001
IMV, *n* (%)	38 (1.74)	48 (1.74)	58 (1.55)	63 (1.33)	107 (1.82)	0.343
NIMV, *n* (%)	15 (0.69)	21 (0.76)	50 (1.34)	74 (1.56)	109 (1.85)	<0.001
LOHS, median (IQR)	8 (11)	7 (10)	6 (9)	6 (8)	6 (7)	<0.001
IHM, *n* (%)	57 (2.61)	113 (4.11)	136 (3.64)	198 (4.18)	293 (4.98)	<0.001

COPD: chronic obstructive pulmonary disease. NA: not available. BMI: body mass index. IMV: invasive mechanical ventilation. NIMV: non-invasive mechanical ventilation. LOHS: length of hospital stay. IHM: in-hospital mortality. *p* value for time trend (Cochran–Armitage tests for binary variables and Jonckheere–Terpstra test for the LOHS).

**Table 4:** Distribution and in-hospital mortality among women and men according to comorbidities, procedures, and in-hospital outcomes among adults hospitalized with sarcoidosis in Spain from 2001 to 2020, according to sex.

	Distribution			In-Hospital Mortality		
Variable	Men	Women	*p*	Men	Women	*p*
Age, mean (SD)	57.09 (16.21)	62.23 (16.01)	<0.001	69.75 (12.91)	72.88 (12.16)	<0.001
Charlson Comorbidity Index, mean (SD)	1.04 (0.92)	0.94 (0.81)	<0.001	1.73 (1.21)	1.56 (1.16)	0.003
Pulmonary sarcoidosis, *n* (%)	8581 (44.47)	9884 (39.69)	<0.001	374 (4.36)	455 (4.6)	0.423
Pneumonia, *n* (%)	1112 (5.76)	1269 (5.1)	0.002	108 (9.71)	116 (9.14)	0.634
COVID 19, *n* (%)	129 (0.67)	155 (0.62)	0.547	20 (15.5)	24 (15.48)	0.996
Respiratory failure, *n* (%)	2939 (15.23)	4212 (16.91)	<0.001	370 (12.59)	481 (11.42)	0.133
COPD, *n* (%)	1684 (8.73)	717 (2.88)	<0.001	109 (6.47)	37 (5.16)	0.212
Asthma, *n* (%)	614 (3.18)	2011 (8.08)	<0.001	12 (1.95)	59 (2.93)	0.193
Acute pulmonary embolism, *n* (%)	202 (1.05)	279 (1.12)	0.460	18 (8.91)	37 (13.26)	0.141
Pulmonary hypertension *n* (%)	883 (4.58)	1638 (6.58)	<0.001	75 (8.49)	118 (7.2)	0.246
Obstructive sleeping apnea *n* (%)	910 (4.72)	591 (2.37)	<0.001	37 (4.07)	18 (3.05)	0.306
Gastroesophageal reflux disease *n* (%)	230 (1.19)	350 (1.41)	0.050	8 (3.48)	12 (3.43)	0.974
Scleroderma, *n* (%)	32 (0.17)	72 (0.29)	0.008	2 (6.25)	6 (8.33)	0.714
Rheumatoid Arthritis *n* (%)	136 (0.7)	363 (1.46)	<0.001	7 (5.15)	15 (4.13)	0.624
Dermatomyositis and Polymyositis, *n* (%)	31 (0.16)	14 (0.06)	0.001	2 (6.45)	1 (7.14)	0.931
Obesity, *n* (%)	1200 (6.22)	2390 (9.6)	<0.001	36 (3)	62 (2.59)	0.482
Tobacco use, *n* (%)	5149 (26.69)	1806 (7.25)	<0.001	186 (3.61)	37 (2.05)	0.001
Oxygen prior to hospital admission, *n* (%)	831 (4.31)	1248 (5.01)	0.001	90 (10.83)	120 (9.62)	0.386
IMV, *n* (%)	314 (1.63)	269 (1.08)	<0.001	136 (43.31)	124 (46.1)	0.500
NIMV, *n* (%)	269 (1.39)	353 (1.42)	0.836	69 (25.65)	69 (19.55)	0.070
LOHS, median (IQR)	6 (9)	6 (8)	0.563	10 (16)	8 (15)	0.290
IHM, *n* (%)	797 (4.13)	1010 (4.06)	0.694	NA	NA	-

COPD: chronic obstructive pulmonary disease. NA: not available. BMI: body mass index. IMV: invasive mechanical ventilation. NIMV: non-invasive mechanical ventilation. LOHS: length of hospital stay. IHM: in-hospital mortality.

**Table 5:** Logistic regression to assess variables associated with IHM among women and men hospitalized with sarcoidosis in Spain from 2001 to 2020, according to sex.

	Women	Men	Both
Variable	OR (95% CI)	OR (95% CI)	OR (95% CI)
20–44 years	1	1	1
45–59 years,	3.21 (2.17–4.76)	2.41 (1.57–3.69)	2.89 (2.16–3.86)
60–74 years	6.76 (4.66–9.82)	4.68 (3.14–6.96)	5.76 (4.38–7.57)
≥75 years	14.25 (9.78–20.78)	10.48 (7.05–15.56)	12.35 (9.38–16.25)
Pneumonia	NS	1.34 (1.07–1.67)	1.25 (1.06–1.48)
COVID 19	3.25 (1.85–5.68)	2.95 (1.76–4.95)	3.15 (2.16–4.6)
Respiratory failure	3.02 (2.58–3.55)	3.07 (2.67–3.54)	2.91 (2.6–3.26)
Asthma	0.52 (0.28–0.98)	0.65 (0.49–0.86)	0.62 (0.48–0.8)
Acute pulmonary embolism	NS	2.79 (1.89–4.12)	2.09 (1.52–2.88)
Obesity	0.6 (0.42–0.87)	0.53 (0.4–0.7)	0.57 (0.45–0.71)
Oxygen prior to hospital admission	NS	NS	1.22 (1.03–1.44)
IMV	16.76 (12.73–22.07)	23.14 (17.36–30.86)	18.93 (15.49–23.12)
NIMV	3.38 (2.4–4.77)	2.6 (1.88–3.59)	2.93 (2.32–3.7)
Men	NA	NA	1.24 (1.11–1.38)

COPD: chronic obstructive pulmonary disease. IMV: invasive mechanical ventilation. NIMV: non-invasive mechanical ventilation. NA: not available: NS: not significant.

## Data Availability

According to the contract signed with the Spanish Ministry of Health and Social Services, which provided access to the databases from the Spanish National Hospital Database (RAE-CMBD, Registro de Actividad de Atención Especializada; Conjunto Mínimo Básico de Datos, Registry of Specialized Health Care Activities; Minimum Basic Data Set), we cannot share the databases with any other investigator, and we have to destroy the databases once the investigation has concluded. Consequently, we cannot upload the databases to any public repository. However, any investigator can apply for access to the databases by filling out the questionnaire available online at http://www.msssi.gob.es/estadEstudios/estadisticas/estadisticas/estMinisterio/SolicitudCMBDdocs/Formulario_Peticion_Datos_CMBD.pdf (accessed on 17 August 2022). All other relevant data are included in the paper.

## Supplementary Materials

The following supporting information can be downloaded at: https://www.mdpi.com/article/10.3390/jcm11185367/s1, Table S1: International Classification of Diseases, Ninth Revision, Clinical Modification (ICD-9-CM) and International Classification of Diseases, Tenth Revision, Clinical Modification (ICD-10-CM) codes used in this investigation; Table S2: Number of hospitalizations and in-hospital mortality of adults with sarcoidosis in first diagnosis position in Spain from 2001 to 2020, according to sex; Figure S1: Number of patients with a code for lung transplant among adults hospitalized with sarcoidosis in Spain from 2001 to 2020; Figure S2: Joinpoint regression to assess time trend in the in-hospital mortality of hospitalized adults with sarcoidosis in Spain from 2001 to 2020, according to sex.
