# Supplementary material for: Sex Differences in Temporal Trends in Hospitalizations and In-Hospital Mortality in Patients with Sarcoidosis in Spain from 2001 to 2020
Source: J Clin Med. 2022 Sep 13;11(18):5367. doi: 10.3390/jcm11185367 (PMC9506482; doi:10.3390/jcm11185367)
Supplement: Supplementary file 1 [file jcm-11-05367-s001.zip › jcm-1897273-supplementary.pdf]

Table S1. International Classification of Diseases, Ninth Revision, Clinical Modification (ICD-9-CM) and International Classification of Diseases, Tenth Revision, Clinical Modification (ICD-10-CM) codes used in this investigation

Diagnosis or procedures	ICD 10 CD	ICD 9 CD
Sarcoidosis (any diagnosis position)	D86XX	135
Pulmonary sarcoidosis	D86.0, D86.1, D86.2	517.8
Pneumonia	J13, J14, J15, J16, J17, J18	481, 482, 483, 484, 485, 486
COVID 19	B34.2, B97.29, U07.1	
Respiratory failure	J96.00, J96.01, J96.02, J96.20, J96.21, J96.22, J96.10, J96.11, J96.12, J96.90, J96.91, J96.92	518.81, 518.83, 518.84,
Chronic Obstructive Pulmonary Disease	J44	491.20, 491.22, 493.20, 493.23, 496
Asthma	J45	493
Acute pulmonary embolism	I26.01, I26.02, I26.09, I26.90, I26.92, I26.93, I26.94, I26.99	415.11, 415.11, 415.12, 415.13, 415.19
Pulmonary hypertension	I270, I27.2X, I27.89	416.0, 416.8, 416.9
Obstructive sleeping apnea	G47.33	327.23
Gastroesophageal reflux disease	K21.0, K21.9	530.11, 530.81
Scleroderma	M34.XX	710.1
Rheumatoid Arthritis	M05.XX, M06.XX	714.0, 714.1, 714.2, 714.81, 714.89, 714.9
Dermatomyositis and Polymyositis	M33.XX	710.3, 710.4
Obesity	E66.0, E66.2, E66.8, E66.9	278.00, 278.01
Tobacco use	Z72.0, Z87.89,F17	305.1, V15.82
Oxygen prior to hospital admission	Z99.81	V46.2
IMV	5A1935Z, 5A1945Z, 5A1955Z'	96.7, 96.70, 96.71, 96.72
NIMV	5A09357, 5A09457, 5A09557, 5A09358, 5A09458, 5A09558	93.90, 93.93, 93.99

Table S2. Number of hospitalizations and in hospital mortality of adults with sarcoidosis in first diagnosis position Spain from 2001 to 2020 according to sex.

			2001/02	2003/04	2005/06	2007/08	2009/10	2011/12	2013/14	2015/16	2017/18	2019/20	Total
Men	First diagnosis position*	N	505	489	515	534	601	588	612	673	857	960	6334
		%	48.89	42.37	40.08	36.43	34.34	29.53	27.03	27.25	30.76	31.02	32.83
	IHM	N	4	6	9	9	17	9	17	11	16	16	114
		%	0.79	1.23	1.75	1.69	2.83	1.53	2.78	1.63	1.87	1.67	1.80
Women	First diagnosis position *	N	599	672	553	608	608	560	583	680	833	1014	6710
		%	40.69	36.84	30.57	28.40	26.73	21.13	19.74	22.43	25.43	29.23	26.95
	IHM	N	15	18	12	9	11	8	6	8	4	15	106
		%	2.50	2.68	2.17	1.48	1.81	1.43	1.03	1.18	0.48	1.48	1.58

* Significant trend $p<0.05$

1.A. Women

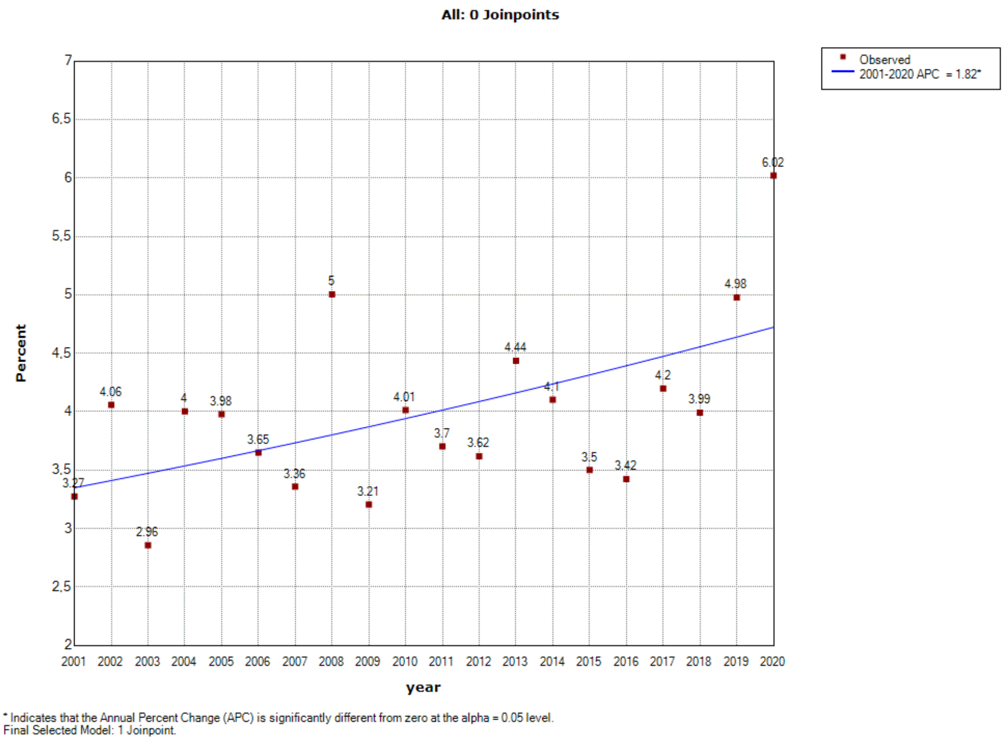

1.B. Men

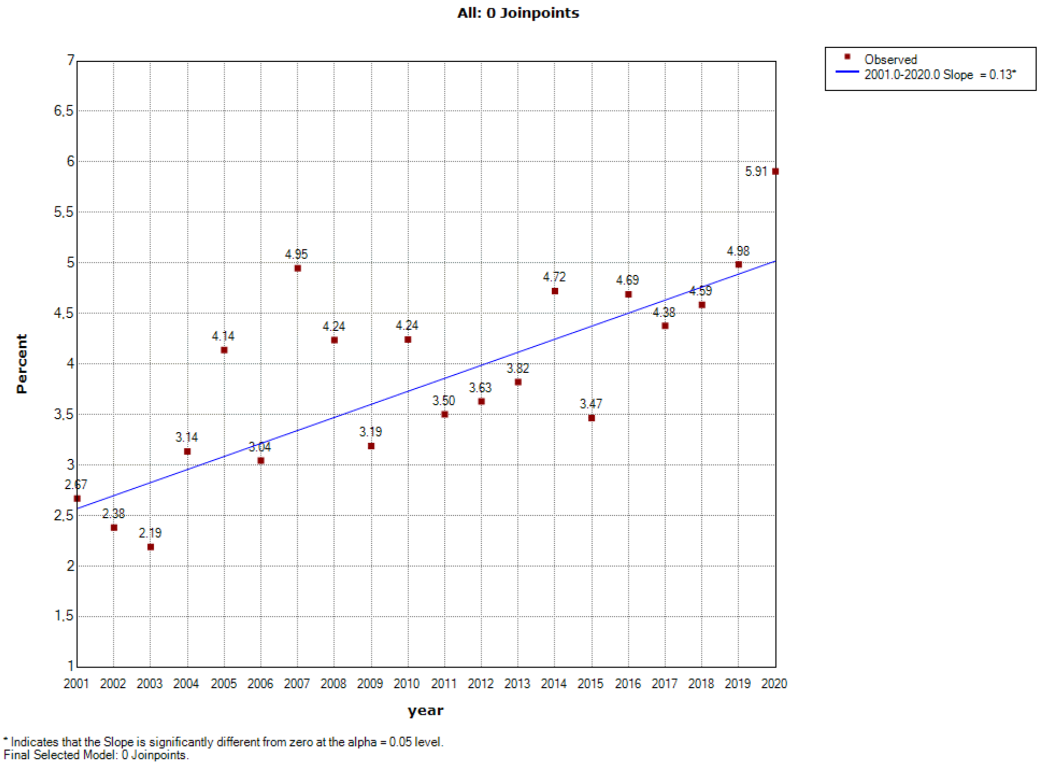

Figure S1. Joinpoint regression to assess time trend in the in-hospital mortality of hospitalizations with sarcoidosis in Spain from 2001 to 2020 according to sex.

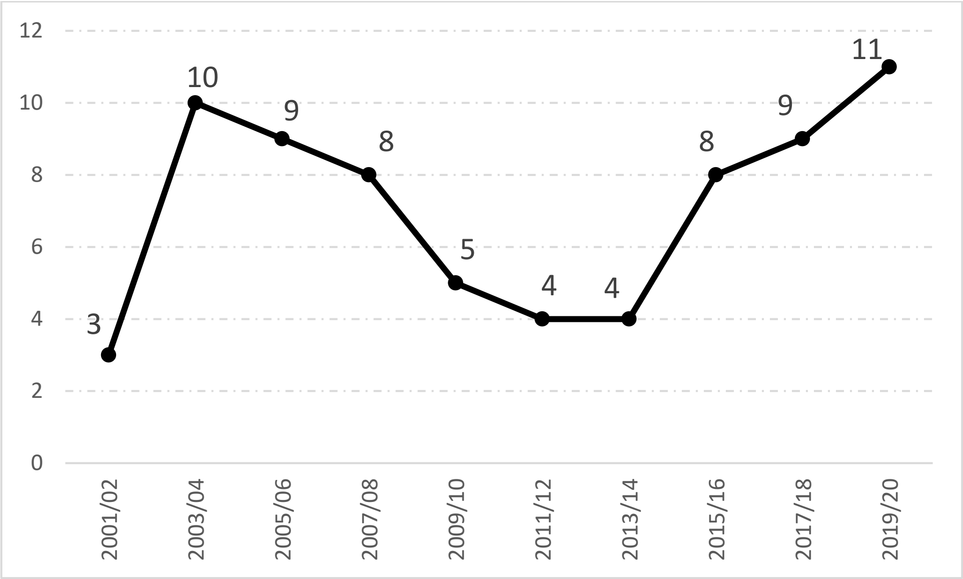

Figure S2. Number of patients with a code for lung transplant among adults hospitalized with sarcoidosis in Spain from 2001 to 2020.
